# Kurzzusammenfassung der neuen S2k-Leitline zu low-grade-muzinösen Neoplasien der Appendix – Was ist wichtig für die klinische Praxis?

**DOI:** 10.1007/s00104-025-02353-5

**Published:** 2025-07-16

**Authors:** Sophie Müller, Carolin Kastner, Christoph-Thomas Germer, Johan Friso Lock, Sven Flemming

**Affiliations:** Klinik und Poliklinik für Allgemein‑, Viszeral‑, Transplantations‑, Gefäß- und Kinderchirurgie, Universitätsklinikum Würzburg, Oberdürrbacherstr. 6, 97080 Würzburg, Deutschland

**Keywords:** Low-grad-muzinöse Neoplasie, LAMN, Appendix, Appendixkarzinom, Pseudomyxoma peritonei, Low-grade mucinous neoplasm, LAMN, Appendix, Appendiceal cancer, Pseudomyxoma peritonei

## Abstract

Mit Erarbeitung und Veröffentlichung der AWMF S2k-Leitlinie „Diagnostik, Therapie und Nachsorge von low-grade-muzinösen Neoplasien der Appendix (LAMN)“ wurde die aktuelle Evidenz in Bezug auf diese seltene Erkrankung zusammengefasst und somit die Grundlage für eine entsprechend evidenzbasierte, onkologische Therapie dieser Patientenkohorte gelegt. Der nachfolgende Artikel stellt eine kurze Zusammenfassung über die wichtigsten Punkte für die klinische Praxis dar.

## Abstract (en)

With the development and publication of the S2k guidelines of the Association of the Scientific Medical Societies in Germany (AWMF) on the diagnostics, treatment and aftercare of low-grade appendiceal mucinous neoplasm (LAMN), the currently available evidence about this rare disease is summarized. The guidelines serve as a foundation for an appropriate evidence-based oncological treatment of this patient cohort. This article provides a short summary of the most important points for the clinical practice.

Die low-grade-muzinöse Neoplasie der Appendix (LAMN) ist eine seltene Tumorerkrankung und wird überwiegend als Zufallsbefund nach Appendektomie mit einer relativen Häufigkeit von 0,3 % diagnostiziert (Abb. 1). Es handelt sich dabei um einen formal noch gutartigen Tumor, der keine Lymphknoten- oder hämatogenen Fernmetastasen ausbildet, jedoch aufgrund der typischen KRAS-Mutation unkontrolliert proliferiert [1]. Darüber hinaus kann es beim Auftreten weiterer Mutationen zur Progression zu einer high-grade-muzinösen Neoplasie der Appendix (HAMN) oder zu einem Adenokarzinom kommen [2]. Je nach pathologischem Tumorstadium und Resektionsstatus kann es bei bis zu 20 % der Patienten mit LAMN im Verlauf weniger Jahre zu einer diffusen intraabdominellen Ausbreitung der Erkrankung im Sinne einer Peritonealkarzinose kommen, welche dann als Pseudomyxoma peritonei (PMP) bezeichnet wird. Somit reicht die Therapie von einer einfachen Appendektomie bis hin zu komplexen multiviszeralen Resektionen (zytoreduktive Chirurgie) mit hyperthermer intraperitonealer Chemotherapie (HIPEC) [3].

**Abb. 1:**
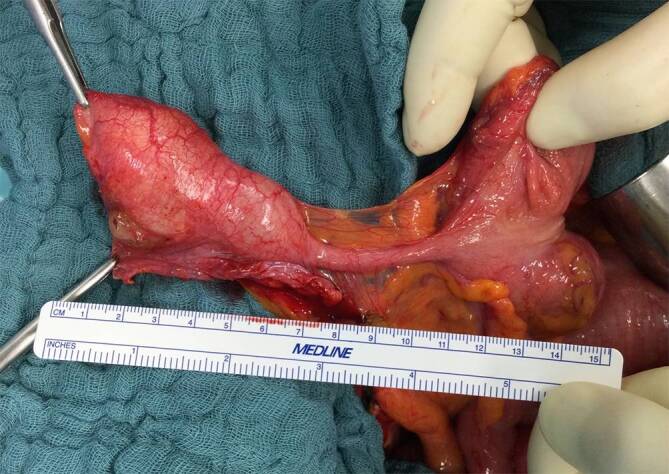
Intraoperativer Befund einer LAMN

Aufgrund der niedrigen Inzidenz der LAMN ist die Erkrankung vielen Ärzten nicht geläufig, und Patienten finden häufig erst verzögert ein geeignetes Behandlungszentrum. Umso wichtiger ist es daher, dass nun mit der erstmalig erarbeiteten und veröffentlichen deutschen AWMF S2k-Leitlinie „Diagnostik, Therapie und Nachsorge von low-grade-muzinösen Neoplasien der Appendix (LAMN)“ [4] ein Behandlungsleitfaden zur Verfügung steht, um auch dieses Patientenkollektiv aus onkologischer Sicht qualitativ hochwertig und auf Grundlage der aktuell verfügbaren Evidenz zu behandeln. Nachfolgend sollen die wichtigsten Aspekte dieser Leitlinie für die alltägliche klinische Praxis adressiert und kommentiert werden.

## Klinisches Erscheinungsbild und Diagnostik

Patienten mit einer LAMN sind häufig asymptomatisch oder präsentieren sich mit einer unspezifischen abdominellen Symptomatik, die auf eine akute Appendizitis oder bei weiblichen Patientinnen auf einen gynäkologischen Fokus hinzuweisen scheint. Das PMP führt initial ebenfalls zu wenig spezifischen Symptomen. Erst bei weit fortgeschrittener Erkrankung zeigt sich durch die progrediente Ansammlung von Muzin eine unspezifische Umfangsvermehrung des Abdomens. In der Diagnosestellung spielen daher die Bildgebung und letztendlich aber die histologische Aufarbeitung eine entscheidende Rolle.

Bei Verdacht auf eine LAMN/PMP in der Computertomographie und/oder Magnetresonanztomographie sollte vor einer weiteren Therapie neben einer kompletten Koloskopie die Bestimmung von der Tumormarkern CEA, CA 19‑9 und CA 125 erfolgen. Dabei dient dies weniger der Diagnosesicherung der Entität LAMN/PMP, sondern der Abgrenzung in Bezug auf andere Tumorentitäten. Die Schnittbildgebung erlaubt bereits eine erste Abschätzung des Ausmaßes der Erkrankung, was im Hinblick auf die Planung des notwendigen operativen Therapieausmaßes und der entsprechenden Komplexität der Patientenaufklärung wichtig ist.

Sowohl bei bildmorphologischem Verdacht als auch nach Zufallsbefundung einer LAMN/PMP im Rahmen operativer Eingriffe wie der Appendektomie sollten die entsprechenden Patienten an ein Zentrum, das in der Therapie dieser Erkrankungsentität erfahren ist, angebunden werden. Die Diskussion der Patientenkasuistik zur Therapieplanung bzw. Festlegung der weiteren Surveillancestrategie im Rahmen einer interdisziplinären Tumorkonferenz ist obligat.

## Chirurgische Therapie der LAMN

**Abb. 2:**
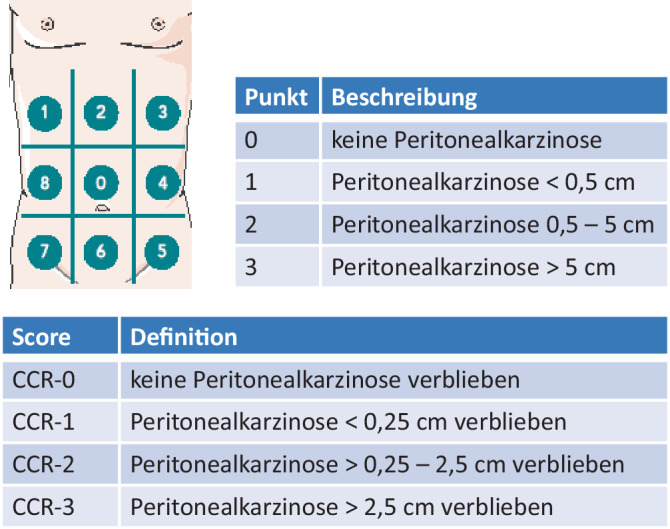
PCI-Score nach Sugarbaker. (Tabellen aus [4])

**Abb. 3:**
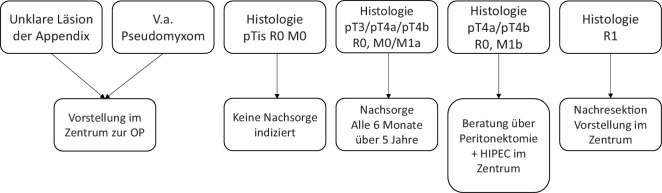
Therapiealgorithmus

Erscheint die Erkrankung in der durchgeführten Bildgebung beschränkt auf die Appendix, ist die diagnostische Laparoskopie mit Appendektomie unter Schnellschnittbedingungen die Therapie der Wahl. Im Rahmen des Eingriffs sollte die vollständige Inspektion der Abdominalhöhle erfolgen, um mögliche Muzinablagerungen außerhalb der Appendix detektieren zu können. Hierbei sollte obligat die Erhebung des Peritonealkarzinose-Index nach Sugarbaker (PCI) erfolgen ([5]; Abb. 2). Bei fehlenden Hinweisen auf extraluminales Muzin sowie einer R0-Resektion der Appendix im Schnellschnitt ist die unmittelbare operative Therapie mit der Appendektomie beendet. Im Rahmen der Appendektomie ist unbedingt darauf zu achten, eine Perforation der Appendix zu vermeiden. Sollte dies laparoskopisch nicht sicher zu gewährleisten sein, ist die Konversion mit nachfolgender offener Appendektomie anzustreben. Für den Fall, dass sich intraoperativ bereits eine Perforation der Appendix mit Muzin der Appendixserosa oder der Mesoappendix aufgelagert (histopathologisch einem pT4a-Tumor entsprechend) zeigt, können mit den Patienten im Vorfeld eine lokale Peritonektomie im rechten Unterbauch sowie eine hypertherme intraperitoneale Chemotherapie (HIPEC) mit Mitomycin C 30 mg/m^2^ für 60 min bei 42 °C als individuelle Maßnahme besprochen werden. Ist dies präoperativ bei intraoperativem Zufallsbefund einer LAMN nicht möglich, kann mit dem Patienten entsprechendes Vorgehen im Rahmen einer operativen Re-Exploration diskutiert werden. Bei pT4a-Befunden (Abb. 3), die R0 reseziert werden können, besteht keine Indikation zur onkologischen Hemikolektomie rechts oder Omentektomie.

## Chirurgische Therapie beim Pseudomyxoma peritonei

Bei bereits bestehendem Verdacht auf ein Pseudomyxoma peritonei ist die explorative Laparotomie mit dem Ziel der vollständigen Zytoreduktion, wenn notwendig, auch im Kontext einer multiviszeralen Resektion mit anschließender HIPEC die Therapie der Wahl. In der Planung des Ausmaßes des chirurgischen Vorgehens gilt insbesondere zu beachten, dass weder die LAMN noch das PMP lymphogen oder hämatogen metastasieren, sodass führend, aber auch ausreichend im Ausmaß der Radikalität der chirurgischen Therapie beider Entitäten die lokale Resektion ist. Das wichtigste Kriterium für das Überleben der Patienten ist in diesem Fall die komplette makroskopische Tumorresektion im Sinne einer CCR-0-Resektion [6]. Daher wird im Rahmen der Leitlinie empfohlen, entsprechende Eingriffe nur an erfahrenen Zentren durchzuführen. Für die Durchführung der HIPEC-Therapie wird durch die aktuelle Studienlage Mitomycin C mit 42 °C über einen Zeitraum von 60 min favorisiert.

## Einfluss der Histopathologie auf die Therapie

Eine einheitliche Klassifikation der histologischen Präparate für Tumoren der Appendix ist erst seit dem Jahr 2010 verfügbar. Wichtig ist v. a. die Abgrenzung zu high-grad-malignen Tumoren oder dem Appendixkarzinom. Die Einteilung sollte anhand der aktuell gültigen TNM-Klassifikation erfolgen. Hierbei ist zu beachten, dass es keine T1- und T2-Tumoren gibt (Tab. 1 und 2).

**Tab. 1:** TNM-Klassifikation, 9. Auflage. (Aus [4])

	Definition
pTis	Tumor auf die Appendix beschränkt mit azellulärem Muzin oder muzinösem Epithel, das bis in die Muscularis propria reichen kann
pT1	Für LAMN nicht vergeben
pT2	Für LAMN nicht vergeben
pT3	Tumorinvasion der Subserosa oder der Mesoappendix
pT4	Tumor perforiert in das viszerale Peritoneum inklusive und oder direkte Invasion anderer Organe oder Strukturen
pT4a	Tumor perforiert viszerales Peritoneum, eingeschlossen muzinöse peritoneale Tumorabsiedlungen innerhalb des rechten unteren Quadranten
pT4b	Tumor wächst direkt in andere Organe oder Strukturen ein
N	n/a
M0	Keine Fernmetastasen
M1	Fernmetastasen
M1a	Intraperitoneal azelluläres Muzin
M1b	Intraperitoneale Metastasen inklusive muzinösem Epithel (zelluläres Muzin)
M1c	Metastasen außerhalb des Peritoneums

**Tab. 2:** UICC-Klassifikation, 9. Auflage. (Aus [4])

Stadium	T‑Kategorie	N‑Kategorie	M‑Kategorie
0	pTis	N0	M0
IIA	pT3	N0	M0
IIB	pT4a	N0	M0
IIC	pT4b	N0	M0
IVA	Jedes T	N0	M1a
IVA	Jedes T	N0	M1b G1
IVB	Jedes T	Jedes N	M1b G2, G3, Gx
IVC	Jedes T	Jedes N	M1c, jedes G

Darüber wird histologisch noch eine Unterscheidung von intraperitonealem, azellulärem Muzin (M1a) von zellulärem Muzin (M1b) vorgenommen. Prinzipiell kann bei einer CCR-0-Resektion eines PMP und dem Vorliegen einer M1a-Situation auf eine HIPEC verzichtet werden. Da der intraoperative Schnellschnitt mit der Bestimmung der Zellularität von Muzin oft Schwierigkeiten hat, ist die Abschätzung der Zellularität des Muzins im Rahmen der Operation keine praktikable Lösung. Mit den Patienten sollte daher bereits präoperativ die Durchführung einer HIPEC in Abhängigkeit des intraoperativen Krankheitsausmaßes besprochen werden.

Bei Patienten, die unter der Verdachtsdiagnose der akuten Appendizitis operiert werden, liegt die definitive Histologie oft erst vor, wenn die Patienten bereits wieder entlassen wurden. Auch hier ist es wichtig, eine pathologische R0-Resektion nachweisen zu können, ansonsten wird die Nachresektion unter Schnellschnittbedingungen empfohlen. Sollte sich Muzin außerhalb der Appendix nachweisen lassen, ist bei azellulärem Muzin (unter Voraussetzung der initialen R0-Resektion) keine erneute Operation mit HIPEC indiziert. Bei Nachweis von zellulärem Muzin außerhalb der Appendix kann mit den Patienten eine erneute operative Exploration mit lokaler Peritonektomie und anschließender HIPEC individuell besprochen werden. Hierbei ist v. a. wichtig, ob im Rahmen des primären Eingriffs makroskopisch Muzin intraabdominell verblieben ist oder nicht. Vor erneuter operativer Exploration sollte immer eine aktuelle Schnittbildgebung durchgeführt werden und eine aktuelle Vorsorgekoloskopie erfolgt sein. Die Operationspräparate und das entnommene Muzin, entnommen sowohl im Rahmen der Primäroperation als auch in einer eventuellen Re-Operation, sollten immer zur histologischen Aufarbeitung eingesendet werden.

## Postoperatives Procedere

Nach Vorliegen der definitiven Histologie ist die (erneute) Diskussion der Patientenkasuistik in einem interdisziplinären Tumorboard an einem entsprechenden Zentrum für die Behandlung dieser Tumorentität empfohlen.

Nach erfolgreicher operativer Therapie im Sinne einer R0-Resektion und CCR-0-Situation wird die Nachsorge für 5 Jahre empfohlen. Im Rahmen dieser sollten die Patienten alle 6 Monate eine Schnittbildgebung erhalten (empfehlenswert ist hier die MRT). Zusätzlich wird die Bestimmung der oben genannten Tumormarker im Rahmen der Kontrollvorstellungen empfohlen. Bei Verdacht auf ein Rezidivgeschehen ist die erneute Vorstellung im Rahmen eines interdisziplinären Tumorboards angezeigt.

## Fazit für die Praxis

- Die LAMN und das PMP präsentieren sich oftmals mit einer unspezifischen Klinik.
- Bei bildmorphologischem Verdacht sollte die Vorstellung an einem Zentrum erfolgen.
- Bei histologischem Zufallsbefund im Rahmen einer Appendektomie sollte ebenfalls die postoperative Vorstellung an einem Zentrum erfolgen.
- Eine onkologische Hemikolektomie rechts ist bei alleiniger LAMN aufgrund der fehlenden lymphogenen Metastasierung nicht notwendig.
